# Carcinoma of an unknown primary: are EGF receptor, Her-2/neu, and c-Kit tyrosine kinases potential targets for therapy?

**DOI:** 10.1038/sj.bjc.6603942

**Published:** 2007-09-18

**Authors:** C Massard, J-J Voigt, A Laplanche, S Culine, A Lortholary, R Bugat, C Theodore, F Priou, M-C Kaminsky, T Lesimple, X Pivot, B Coudert, J-Y Douillard, Y Merrouche, K Fizazi

**Affiliations:** 1 Department of Medicine, Institut Gustave Roussy, 39 rue Camille Desmoulins, Villejuif 94805, France; 2 Department of Pathology, Centre Claudius Regaud, Toulouse, France; 3 Department of Statistics, Institut Gustave Roussy, Villejuif, France; 4 Department of Medicine, Centre Val d'Aurelle, Montpellier, France; 5 Department of Medicine, Centre Paul Papin, Anger, France; 6 Department of Medicine, Institut Claudius Regaud, Toulouse, France; 7 Department of Medicine, Centre Hospitalier La Roche/Yon, La Roche/Yon, France; 8 Department of Medicine Centre Alexis Vautrin, Nancy, France; 9 Department of Medicine, Centre Eugène Marquis, Rennes, France; 10 Department of Medicine, Centre Hospitalier Universitaire, Besançon, France; 11 Department of Medicine, Centre G.F. Leclerc, Dijon, France; 12 Department of Medicine, Centre René Gauducheau, Nantes, France; 13 Department of Medicine, Institut de Cancérologie de la Loire, Saint-Etienne, France

**Keywords:** carcinoma of an unknown primitive, tyrosine kinase receptor, EGFR, c-Kit, Her-2/neu overexpression

## Abstract

Carcinomas of an unknown primary site (CUP) are heterogeneous tumours with a median survival of only 8 months. Tyrosine kinase inhibitors are promising new drugs. The aim of this study was to determine the expression of EGF-receptor, Her-2/neu, and c-Kit tyrosine kinases in CUP. Paraffin-embedded specimens were obtained from 54 patients with a CUP who were included in the GEFCAPI 01 randomised phase II trial. Immunohistochemistry was performed using the Dako autostainer with antibodies directed against HER-2/neu protein, EGFR protein, and c-Kit protein (CD117). EGFR expression was found in 36 out of 54 samples (66%). In contrast, Her-2/neu overexpression and c-Kit positivity were only detected in 4 and 10% of patients, respectively. No significant association was found between the expression of the tyrosine kinase receptors and prognosis. EGFR expression was significantly associated with response to cisplatin-based chemotherapy: the response rates were 50 and 22% in patients with EGFR-positive tumours and EGFR-negative tumours, respectively (*P*<0.05). This study shows that EGFR is frequently expressed in CUP. This finding may prompt clinical trials investigating EGFR inhibitors in this setting. In contrast, c-Kit expression and Her-2/neu overexpression occur infrequently in CUP. EGFR expression was correlated to tumour chemosensitivity.

Carcinoma of an unknown primary site (CUP) rank among the 10 most frequent cancers worldwide and its prognosis is notoriously poor with median survival rates attaining 8 months (Pavlidis and Fizazi, 2005). Carcinoma of an unknown primary site are heterogeneous tumours whose origin is unidentifiable at the time of the diagnosis but they share the unique clinical feature of metastatic disease: a slow local development and a high metastatic potential. Despite advances in tumour imaging and pathology, patients with CUP still account for about 5% of all cancer patients. The primary site remains frequently unknown but necroptic studies indicate that the primary sites are most often the pancreas, lung, gut, and kidney.

The treatment of patients presenting with a CUP remains a daily challenge for physicians. Although systemic chemotherapy is usually recommended, the optimal regimen remains to be determined. To improve the poor outcome of patients, several recent studies have focused on the introduction of new cytotoxic agents such as gemcitabine (GC), irinotecan (IC), and taxanes which exhibit a broad spectrum of clinical activity (Greco *et al*, 2002; Culine *et al*, 2003). However, no randomised trial has provided a clear evidence of a survival benefit for CUP patients (Fizazi, 2006).

In addition, the biology of CUP is still very poorly understood (Fizazi, 2006). Growth factor receptors with tyrosine kinase activities such as EGFR, HER-2/neu, and c-Kit have recently emerged as promising targets for novel therapeutic agents, especially in some poorly chemosensitive neoplasms (Krause and Van Etten, 2005). This prompted us to study receptor tyrosine kinases, which could be potential targets for novel therapies in patients with CUP.

We therefore studied tumour specimens from 54 patients with CUP enrolled in one of a large prospective randomised phase II trial (Culine *et al*, 2003) to determine EGFR, Her-2/neu, and c-Kit protein expression.

## MATERIALS AND METHODS

### Patients

Tissues were obtained from the original biopsy specimens of 54 patients who were enrolled in a randomised phase II trial (GEFCAPI 01) conducted by the French Study Group of Carcinomas of an unknown primary from August 1999 to November 2000 (Culine *et al*, 2003). The study was approved by the Ethics Committee in Montpellier. All patients signed a written informed consent before participation. Patients were randomly assigned to one of the two treatment regimens at the time of study entry and could receive chemotherapy combinations including cisplatin with GC or IC. Patients were excluded if they had any of the following features: a CUP subset requiring specific treatment (i.e. women with adenocarcinoma with axillary lymph node involvement alone, women with papillary serous carcinoma of the peritoneum, patients with squamous carcinoma exclusively involving cervical or inguinal lymph nodes, carcinomas with neuroendocrine features, and patients with carcinoma in a potentially resectable site) and those with symptomatic brain metastasis. To exclude other malignancies (lymphoma, melanoma, sarcoma, and neuroendocrine carcinoma), immunoperoxidase staining with antibodies against leukocyte common antigen, cytokeratin, neuroendocrine markers (chromogranin and synaptophysin), and melanoma markers (S-100 protein and homatropine methylbromide-45) was recommended in poorly differentiated carcinoma. All specimens were centrally reviewed by a single pathologist (JJV). Well-differentiated adenocarcinoma was the most common histologic type. The dominant visceral sites of disease were the bone, lung, and liver, whereas the mediastinum was the most common site of lymph node involvement. The results of this clinical trial have already been published (Culine *et al*, 2003). The study demonstrated the activity of both regimens in CUP patients. Response to chemotherapy we evaluated by CT scan after every two cycle of chemotherapy and independent radiologic review was carried out. Moreover, a simple prognostic model was established with Performans Status and LDH serum in CUP patients (Culine *et al*, 2002).

### Immunohistochemistry

Formalin-fixed, paraffin-embedded sections were immunostained for EGFR, HER-2/neu, and c-Kit. Five-micrometer sections were cut, deparaffinised, and rehydrated. There is no SOP (Standardized Operation Procedures) for tissue collection. The slides were processed using an autostainer (Dako, Carpinteria, CA, USA) with a streptavidin–biotin complex and diaminobenzidine as the chromogens. The antibodies used were the PharmDx EGFR kit (Dako), the polyclonal rabbit anti-human-c-Kit (CD117, an epitope of KIT) (A4502, Dako) (dilution 1 : 50) and the polyclonal rabbit anti-human HER-2/neu – c-erbB-2 (A0485, Dako) (dilution 1 : 1500) in one slide for each antibody.

Microwave oven antigen retrieval from tissue sections was only performed for HER-2/neu. For EGFR staining, tissue sections were digested with protease K at 21°C for 5 min. Nonspecific binding sites were bound using Protein Block (Dako). The slides were incubated with primary antibody for 60 min (HER-2/neu and c-Kit) and 30 min for EGFR. Staining was revealed using the following chromogens: biotinylate secondary antibody, peroxidase-labelled streptavidin and 3,3′-diaminobenzidine tetrahydrochloride-hydrogen peroxide.

Dilution and incubation periods were optimised for each antibody by using positive controls. Specimens whose incubation with the primary antibody had been omitted were used as a negative control. In each experiment, tumours with defined alterations in EGFR, HER-2/neu, or c-Kit were used as positive controls. Each case was scored by a pathologist blinded to patient identity, and scoring system for EGFR, Her2/neu and Kit is based on College of American Pathologists Cell Markers Committee guidelines (Arch Pathol Lab Med 2002–2006).

EGFR positivity revealed by chromogenic staining was localised mainly in the cell membrane. Three staining categories were defined before assessing EGFR: specimens with no or less than 1% of positive cells were defined as ‘negative’, specimens with 1–20% of positive cells were defined as ‘positive ++’ and specimens with more than 20% of positive cells were defined as ‘positive +++’. These definitions of positive and negative results are in accordance with the published literature (Goldstein and Armin, 2001) but may require modification in specific context.

HER-2/neu expression was confirmed when staining was localised in the membrane. Specimens were scored as 0 (no staining or less than 10% of positive cells), 1+ (weak and focal staining of more than 10% of positive cells), 2+(weak to moderate and complete membrane staining of more than 10% of positive cells), and 3+ (strong and complete membrane staining of more than 10% of positive cells). Specimens scored as 3+ are Her-2/neu overexpression.

C-Kit expression was scored as negative when there was no staining for c-Kit or when staining was observed in less than 1% of the cells and as positive when the cytoplasm was strongly stained with or without membrane staining in 10% or more cancer cells. C-Kit expression was controlled with mast cells known to express c-Kit.

### Statistical analysis

Fischer's exact test was used to evaluate differences in clinical characteristics between positive and negative EGFR, Her-2/neu, and c-Kit expression.

The following variables were studied: age, sex, pathology review, ECOG, serum LDH, disease site, chemotherapy, and response to chemotherapy. Differences in survival duration between subgroups were analysed using a two-sided log rank.

All *P*-values were two-sided. *P*<0.05 was considered statistically significant. All analyses were carried out with the SPSS package (SPSS program stat).

## RESULTS

Fourteen centres enrolled 80 patients onto the GEFCAPI 01 trial. A pathological review of 75 specimens was performed. Tissue from the original biopsy specimens was available in 54 patients for the present immunohistochemical analysis. Patient characteristics are summarised in Table 1.

Eighteen (33%) cases showed no EGFR expression. EGFR was expressed in 36 (66%) of 54 available samples, displaying moderate staining in 17 (31%) cases and strong EGFR positivity in 19 (35%) cases.

In the univariate analysis, EGFR expression correlated with response to chemotherapy (Table 2), but not with age, gender, tumour site, and pathological differentiation. Response to cisplatin-based chemotherapy was 50 and 22% in patients with EGFR-positive and -negative tumours respectively (*P*=0.046).

Her-2 overexpression was found in only 2 (4%) of 54 tumour specimens. There was no statistically significant correlation between Her-2/neu staining and clinical characteristics, as summarised in Table 1.

Only six cases (10%) showed at least some c-Kit expression. There was no statistically significant correlation between c-Kit staining and clinical characteristics, as summarised in Table 1.

## DISCUSSION

In the present study, we showed that EGFR is expressed in a majority (66%) of CUP. Moreover, EGFR expression correlated with response to cisplatin-based chemotherapy. This study is relatively small but it is one of the largest randomised trials in CUP. Treating patients with CUP remains a challenge for physicians and a better understanding of the biology of this neoplasm with the identification of reliable tumour markers that reflect tumour aggressiveness or of predictive factors for new therapeutics might lead to targeted therapies. Growth factor receptors like EGFR, HER-2, and c-Kit have recently emerged as promising targets for novel therapeutic agents in malignancies such as lung cancer (Shepherd *et al*, 2005), breast cancer (Slamon *et al*, 2001), and gastrointestinal stromal tumour (GIST) (Heinrich *et al*, 2002). This study provides evidence for tyrosine kinase receptor expression/lack of expression in CUP and may allow the study of targeted therapies in these patients. Moreover, immunohistochemistry is an indispensable tool in diagnosis and management of CUPs. The role of pathologist is to determine the histopathological type rather than the origin of the primary tumour in view of a suitable therapy based on clinical, morphological, and phenotypic data (Fizazi, 2006).

In the last decade, EGFR has emerged as one of the most important targets for drug development in oncology. Because EGFR is expressed and associated with poor prognosis and a more malignant phenotype in many neoplasms, it has been investigated as a potential target for cancer therapy (Baselga and Arteaga, 2005; Mendelsohn and Baselga, 2006). There are currently two treatment options using anti-EGFR agents under clinical development: a monoclonal antibody directed at the extracellular domain of the receptor and small molecule inhibitors of the EGFR tyrosine kinase. Clinical trials with a humanised murine chimeric monoclonal antibody to EGFR (C225, Cetuximab) in combination with chemotherapy or radiotherapy have shown significant clinical activity in advanced colorectal carcinomas (Cunningham *et al*, 2004) squamous cell carcinomas of the head and neck (Vermorken *et al*, 2007), and non-small cell lung cancer (NSCLC) (Janne *et al*, 2004; Giaccone, 2005). On the other hand, a large number of inhibitors of the EGFR tyrosine kinase are active against NSCLC (Thatcher *et al*, 2005) and pancreatic cancer. It is well known that lung cancer is one of the most frequent origin of unknown primary cancer (Fizazi, 2006). The BR.21 study evaluated the effectiveness of erlotinib in second-line treatment in NSCLC. The results of BR.21 study demonstrated that erlotinib has the potential to improve overall survival to unselected chemotherapy refractory NSCLC patients (Shepherd *et al*, 2005). A subset of NSCLS patients achieve impressive responses with TKI. Several authors have showed that the mutation or amplification of EGFR are associated with dramatic and sustained response to TKI in lung cancer (Taron *et al*, 2005; Tsao *et al*, 2005; Shigematsu and Gazdar, 2006). Compounds such as cetuximab, gefitinib, and erlotinib could prove valid for targeting tumours expressing EGFR in patients selected according to this marker and may represent a novel therapeutic strategy in patients with CUP (Sequist *et al*, 2007). There is a need for strategies with anti-EGFR agents alone or in combination with conventional chemotherapies and to explore combinations with other molecularly targeted therapies such as angiogenesis inhibitors. These new therapeutic approaches may represent an exciting and promising way of improving the unfavourable prognosis in CUP patients.

Response to cisplatin-based chemotherapy was better in patients with EGFR-positive as compared to those with EGFR-negative tumours whereas there is no difference in overall survival. Several studies suggest that while gefitinib or erlotinib are not effective in the general NSCLC population (Shepherd *et al*, 2005), these targeted therapies have activity in selected patients, and never-smokers and patients of Asian origin and women with adenocarcinoma might expect improvement in survival (Brown and Shepherd, 2005). Moreover, EGFR mutation or EGFR expression may be a positive prognostic factor for survival in advanced NSCLC patients treated with chemotherapy with or without erlotinib, and may predict greater likelihood of response to chemotherapy with or without erlotinib. However, our results must be viewed cautiously since the number of patients was relatively small.

A study by Hainsworth *et al* (2000) showed that 11% of tumour specimens overexpressed Her-2/neu using the Dako immunohistochemical method in a large group of patients who had received uniform treatment with cisplatin-based chemotherapy. In that study, Her-2/neu overexpression occurred mainly in patients with poorly differentiated carcinoma or patients whose tumour was predominantly located above the diaphragm, in the mediastinum or lungs. Another study demonstrated that patients with CUP have a high overexpression of Her-2/neu (Pavlidis *et al*, 1995). In our study, Her-2/neu overexpression was observed in 4% of tumour specimens but 60% of patients had adenocarcinoma and only 30% patients had poorly undifferentiated adenocarcinoma and undifferentiated carcinoma. In these studies, the difference in Her-2/neu overexpression could be explained by the patient population (poorly differentiated *vs* well-differentiated carcinoma). Moreover, it might be useful to standardise staining procedures used or to compare Her-2/neu overexpression with an immunohistochemical test and FISH amplification.

C-Kit is a tyrosine kinase receptor, which is a target for imatinib mesylate (Gleevec; Novartis Pharma, Basel, Switzerland). Imatinib therapy of c-Kit-positive tumours is an example of rationally targeted cancer therapy, like trastuzumab for the treatment of Her-2/neu-positive breast cancer. Certain malignancies such as chronic myeloid leukaemia (O'Hare *et al*, 2006) and GISTs (Joensuu *et al*, 2002) express c-Kit and respond favourably to imatinib therapy. In a recent study (Went *et al*, 2004), c-Kit expression was shown to occur infrequently in most tumour types. In our study, only 11% of CUP expressed c-Kit, and its expression was not correlated with any clinical patient characteristics.

**Figure 1:**
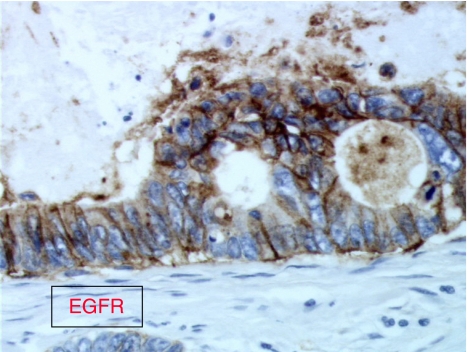
Immunohistochemical patterns of EGFR expression in a carcinomas of an unknown primary site (CUP) specimen showing strong EGFR immunostaining (magnification × 400).

**Figure 2:**
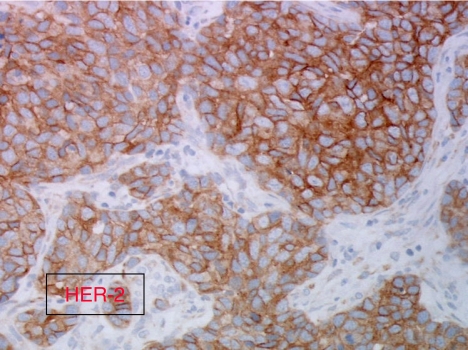
Immunohistochemical patterns of Her-2/neu expression in a carcinomas of an unknown primary site (CUP) specimen showing strong HER-2/neu immunostaining (magnification × 400).

**Figure 3:**
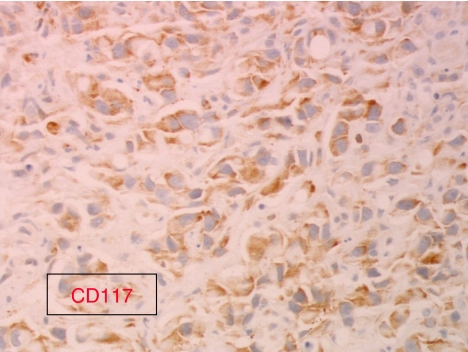
Immunohistochemical patterns of c-Kit expression in a carcinomas of an unknown primary site (CUP) specimen showing strong c-Kit immunostaining (magnification × 400).

Anti-EGFR agents may be evaluated in patients with CUP, which overexpress EGFR immunohistochemically. It would be interesting to also study EGFR mutations and EGFR amplification in tumour samples from these patients. Tumour tissues from GEFCAPI 01 are not available to study other molecular markers such as Her-2 amplification, c-Kit mutations, or EGFR mutations or amplification. This analysis could be performed on another clinical trial and offers more extensive information for selecting targeted therapies. In particular, in lung cancer EGFR mutations or EGFR amplification could predict response to EGFR tyrosine kinases inhibitors (Shigematsu and Gazdar, 2006). Anti-EGFR agents may be evaluated in second- or first-line therapy, in combination with chemotherapy or alone (Figures 1, 2 and 3).

## Figures and Tables

**Table 1:** Clinical and pathological characteristics of patients with and without Her-2/neu overexpression, c-Kit expression and EGFR expression

**Characteristics**	**All patients (*n*=54)**	**EGFR− (*n*=18)**	**EGFR+ (*n*=36)**	**HER2/neu− (*n*=52)**	**HER2/neu+ (*n*=2)**	**c-KIT− (*n*=48)**	**c-KIT+ (*n*=6)**
*Age (years)*							
Median	58	58	55.5	58	52	55.5	66.5
Range	33–73	33–68	41–73	33–73	45–59	33–73	48–71
							
*Sex*							
Male	31 (57%)	10 (55%)	21 (58%)	29 (56%)	2 (100%)	29 (60%)	2 (33%)
Female	23 (43%)	8 (45%)	15 (42%)	23 (44%)	0	19 (40%)	4 (67%)
							
*Pathology*							
Adenocarcinoma	29 (53%)	10 (55%)	19 (53%)	27 (52%)	2 (100%)	27 (56%)	2 (33%)
Poorlydifferentiated	18 (33%)	7 (38%)	11 (30%)	18 (35%)	0	16 (33%)	2 (33%)
Undifferentiated	7 (13%)	1 (5%)	6 (17%)	7 (13%)	0	5 (11%)	2 (33%)
							
*Treatment*							
1	28 (51%)	7 (38%)	21 (58%)	27 (52%)	1 (50%)	27 (56%)	1 (17%)
2	26 (49%)	11 (62%)	15 (42%)	25 (48%)	1 (50%)	21 (44%)	5 (83%)
							
*Serum LDH*							
Normal	26 (48%)	9 (50%)	17 (47%)	26 (50%)	0	23 (48%)	3 (50%)
Elevated	22 (40%)	8 (45%)	14 (39%)	21 (40%)	1	19 (40%)	3 (50%)
Not available	6 (12%)	1 (5%)	5 (14%)	5	1	6	0
							
*ECOG PS*							
0	43 (79.5%)	14 (78%)	29 (80%)	41 (79%)	2	38 (79%)	5 (83%)
1	10 (18.5%)	4 (22%)	6 (17%)	10 (19%)	0	9 (19%)	1 (17%)
Not available	1		1	1	0	1	0
							
*Tumour site*							
Lymph nodes	32 (60%)	10 (55%)	25 (70%)	35 (67%)	2 (100%)	36 (75%)	4 (66%)
Liver	27 (50%)	11 (60%)	16 (44%)	27 (52%)	0	25 (52%)	2 (33%)
Lung	28 (52%)	11 (60%)	17 (47%°	26 (50%)	2 (100%)	26 (54%)	2 (33%)
Bone	19 (35%)	9 (50%)	10 (28%)	18 (35%)	1 (50%)	18 (37%)	0
Peritoneum	3 (5%)	3 (16%)	9 (25%)	3 (6%)	0	3 (6%)	0
Adrenal gland	6 (11%)	1 (5%)	5 (14%)	12 (23%)	0	6 (12%)	0
Pleura	4 (7%)	1 (5%)	3 (8%)	4 (8%)	0	4 (8%)	0
Brain	2 (4%)	0	2 (5%)	2 (4%)	0	1 (2%)	1 (17%)
Cutaneous	1 (2%)	0	1 (2.5%)	1 (2%)	0	1 (2%)	0
Others	10 (18%)	4 (22%)	6 (17%)	10 (19%)	0	10 (21%)	0

EGFR-negative tumour patient: EGFR score 1+.

EGFR-positive tumour patient: EGFR score 2+ or 3+.

Treatment 1: cisplatin and gemcitabin.

Treatment 2: cisplatin and irinotecan.

**Table 2:** EGFR expression and response to chemotherapy

**Response**	**EGFR negative (*n*=18)**	**EGFR positive (*n*=36)**	***P*-value**
Complete response	0	1 (3%)	
Partial response	4 (22%)	17 (47%)	
Stable disease	5 (28%)	9 (25%)	
Progressive disease	7 (39%)	5 (14%)	
Not evaluable	2 (1%)	4 (11%)	
Objective response rate	4 (22%)	18 (50%)	<0.05

EGFR-negative tumour patient: EGFR score 1+.

EGFR-positive tumour patient: EGFR score 2+ or 3+.
