# Carcinoma of an unknown primary: are EGF receptor, Her-2/neu, and c-Kit tyrosine kinases potential targets for therapy?

**DOI:** 10.1038/sj.bjc.6604800

**Published:** 2008-11-25

**Authors:** C Massard, J-J Voigt, A Laplanche, S Culine, A Lortholary, R Bugat, C Theodore, F Priou, M-C Kaminsky, T Lesimple, X Pivot, B Coudert, J-Y Douillard, Y Merrouche, K Fizazi

**Correction to**: *British Journal of Cancer* (2008) **97,** 857–861, doi:10.1038/sj.bjc.6603942

The authors of the above paper, first published in October 2007, would like to add the following acknowledgement to their paper:

We thank Programme Hospitalier de Recherche Clinique (PHRC).

